# Assessing whole-exome sequencing data from undiagnosed Brazilian patients to improve the diagnostic yield of inborn errors of immunity

**DOI:** 10.1186/s12863-023-01137-2

**Published:** 2023-06-30

**Authors:** Cristina Santos Ferreira, Ronaldo da Silva Francisco Junior, Alexandra Lehmkuhl Gerber, Ana Paula de Campos Guimarães, Flávia Anisio Amendola, Fernanda Pinto-Mariz, Monica Soares de Souza, Patrícia Carvalho Batista Miranda, Zilton Farias Meira de Vasconcelos, Ekaterini Simões Goudouris, Ana Tereza Ribeiro Vasconcelos

**Affiliations:** 1 Bioinformatics Laboratory-LABINFO, National Laboratory of Scientific Computation LNCC/MCTIC, Av. Getúlio Vargas, 333, Quitandinha, Petrópolis, Rio de Janeiro 25651-075 Brazil; 2 Allergy and Immunology Service of Institute of Women, Children and Adolescents’ Health Fernandes Figueira (IFF) - Oswaldo Cruz Foundation (FIOCRUZ), Rio de Janeiro, RJ Brazil; 3 Allergy and Immunology Service of the Martagão Gesteira Institute for Childcare and Pediatrics (IPPMG), Federal University of Rio de Janeiro (UFRJ), Rio de Janeiro-RJ, Brazil; 4 Federal Hospital for State Employees (HFSE)–Health Ministry, Rio de Janeiro, RJ Brazil; 5 Lagoon Federal Hospital (HFL)–Health Ministry, Rio de Janeiro, RJ Brazil; 6 Laboratory of High Complexity of the Institute of Women, Children and Adolescents’ Health Fernandes Figueira (IFF) - Oswaldo Cruz Foundation (FIOCRUZ), Rio de Janeiro, RJ Brazil

**Keywords:** Whole exome sequencing, Single nucleotide variants, Monogenic disorder, Inborn errors of immunity

## Abstract

**Objectives:**

Inborn error of immunity (IEI) comprises a broad group of inherited immunological disorders that usually display an overlap in many clinical manifestations challenging their diagnosis. The identification of disease-causing variants from whole-exome sequencing (WES) data comprises the gold-standard approach to ascertain IEI diagnosis. The efforts to increase the availability of clinically relevant genomic data for these disorders constitute an important improvement in the study of rare genetic disorders. This work aims to make available WES data of Brazilian patients’ suspicion of IEI without a genetic diagnosis. We foresee a broad use of this dataset by the scientific community in order to provide a more accurate diagnosis of IEI disorders.

**Data description:**

Twenty singleton unrelated patients treated at four different hospitals in the state of Rio de Janeiro, Brazil were enrolled in our study. Half of the patients were male with mean ages of 9 ± 3, while females were 12 ± 10 years old. The WES was performed in the Illumina NextSeq platform with at least 90% of sequenced bases with a minimum of 30 reads depth. Each sample had an average of 20,274 variants, comprising 116 classified as rare pathogenic or likely pathogenic according to American College of Medical Genetics and Genomics and the Association (ACMG) guidelines. The genotype-phenotype association was impaired by the lack of detailed clinical and laboratory information, besides the unavailability of molecular and functional studies which, comprise the limitations of this study. Overall, the access to clinical exome sequencing data is limited, challenging exploratory analyses and the understanding of genetic mechanisms underlying disorders. Therefore, by making these data available, we aim to increase the number of WES data from Brazilian samples despite contributing to the study of monogenic IEI-disorders.

## Objective

Inborn errors of Immunity (IEI) are a broad group of monogenic inherited disorders often caused by deleterious germline variants, comprising 485 illnesses identified up to date with heterogeneous phenotypic features that lead to overlapping clinical manifestations and misdiagnosis [1–3]. Advances in massively parallel sequencing technologies, such as whole exome sequencing (WES), and whole genome sequencing (WGS) have enabled much better resolution of various IEI disorders since a broader screening to identify new disease-related genes is possible [4–7]. Considering the growing number of genes associated with IEI, exploring publicly available samples may improve the diagnostic yield of these disorders contributing to the ongoing construction of a genetic background of IEI. However, until November 2022, a few WES data from Brazilian patients were available in the National Center of Biotechnology Information (NCBI) Sequence Read Archive (SRA) (https://www.ncbi.nlm.nih.gov/sra/). Most of the publicly available data in repositories originated from samples with assertive genetic diagnosis, usually achieved through identifying pathogenic or likely pathogenic single nucleotide variants (SNVs) and insertion or deletion variants (INDELs). Data sharing may contribute to a convergent prioritization of variants, besides improving the criteria for classifying deleterious variants. Such achievement is particularly important in identifying of new genes related to monogenic disease [8]. In this context, we aimed to provide the WES from undiagnosed Brazilian patients suspicious of IEI available in NCBI/SRA database to improve the genetic diagnosis of monogenic disorders, variant prioritization and classification strategies and facilitating the access to Brazilians massively parallel sequencing data (see Data Set 1) [9].

## Data description

We conducted a genetic screening of WES data from 20 singleton unrelated patients with suspicion of IEI treated by the Brazilian public Unified Health System (“Sistema Único de Saúde” or SUS) admitted from June 2017 to April 2018 to different medical centers in Rio de Janeiro. Seven patients were admitted to the Instituto de Puericultura e Pediatria Martagão Gesteira (IPPMG) of the Universidade Federal do Rio de Janeiro (UFRJ), eight from the Serviço de Alergia e Imunologia, of the Instituto Fernandes Figueira (IFF) in the Fundação Oswaldo Cruz (FIOCRUZ), four from the Hospital Federal dos servidores do Estado (HFSE) of the Health Ministry, and one from Hospital Federal da Lagoa (HFL) of the Health Ministry. All participants were evaluated by a medical expert team. Still, the limited availability for performing some immunological tests, and discontinuity in the patient follow-up were a challenge in their in-depth phenotypic background.

Our cohort included 10 males and 10 females with overall mean ages of 11 ± 7 years old (age is not available for eight patients) (Data Table 1) [10]. Two patients have a family history of IEI. Patient 17 has a son who carries a likely pathogenic variant related to Wiskott-Aldrich Syndrome (manuscript submitted for publication), and patient 9 has a grandfather reported with Agammaglobulinemia phenotype. However, we have not identified disease-causing variants in our patients to confirm the same phenotype. All subjects and their guardians agreed to participate in this study by signing an informed written Ethical Consent Form approved by The Institutional Ethical Committee from the Instituto Fernandes Figueira study protocol (no. CAAE42934815.4.0000.52695269), and the Ethical Committee of the Instituto Nacional do Câncer (153/10). Furthermore, we safeguard the exclusivity of the patient’s personal information to researchers and clinicians who developed this study. Thus, all publicly accessible patient’s data were de-identified before publication preventing identification by third parties during secondary analysis.

Genomic DNA was extracted from peripheral blood lymphocytes taken from each patient using the QIAmp DNA Mini Kit® (QIAGEN®) according to the manufacturer’s instructions. The WES libraries were prepared using Illumina TruSeq® Exome Kit (8 rxn × 6plex) according to the manufacturer’s protocol. The Illumina NextSeq® 500/550 High Output Kit v2 (150 cycles) was used, generating 2 × 75 bp paired-end reads to provide the sequencing data. The raw data files in FASTQ format were processed in 2022 using an in-house bioinformatic pipeline previously described by us [11–14]. Our framework includes reads mapping, quality control, and variant calling and annotation. We used fastqc (http://www.bioinformatics.babraham.ac.uk/projects/fastqc/) and Trimmomatic [15] to inspect the quality of sequences generated and remove bad-formed reads. The remaining sequences were mapped to the human reference genome (GRCh38) using Bowtie2 version 2.3.5.1 [16, 17]. Additional BAM file analysis was performed with Samtools version 1.11 [18] for sorting and mapping quality filtration (Q30). Duplicate reads were marked using Picard MarkDuplicates tool version 2.20.7 (http://broadinstitute.github.io/picard). Using Genome Analysis Toolkit (GATK) software version 4.1.20 [19], we recalibrated the base quality of BAM files using Base Quality Score Recalibration (BQSR) steps followed by variant calling in the HaplotypeCaller tool. To annotate the genetic consequences, populational allele frequencies, molecular impact, and effects of the variants identified in our analysis, we used SnpEff and SnpSift software version 5.0 [20, 21]. The resulting variants are available in NCBI/dbSNP database (see Data Set 2) [22].

About 20% of sequencing reads were filtered out after quality control steps. On average, 90% of exonic bases covered by the probes had at least 30 reads (see Data Table 2) [23]. The variant classification strategy was based on the guidelines of the American College of Medical Genetics and Genomics and the Association for Molecular Pathology (ACMG/AMP) [24]. To further automate the classification analysis, we used the VarSome clinical database to assign the ACMG/AMP criteria. The filtering approach is shown in data file 1 [25]. We identified a total of 65,700 SNVs and INDELs during variant calling with a mean of 20,274 variants per sample (Data Set 2; Data File 1) [22, 25]. The molecular consequences of the SNVs identified include missense variants (32.5%), synonymous variants (32%); nonsense variants (28.8%); splicing site variants (4.5%), truncating variants (1.3%), inframe variants (0.7%) (see Data File 1) [25]. To select potential pathogenic variants, we focused our analysis on rare (minor frequency allele ≤ 0.01) protein-altering variants, including truncating variants (stop gain/loss, start loss, or frameshift), missense variants, canonical splice-site variants, in-frame insertions and deletions, and indels. We used two approaches to select qualifying variants. First we included VarSome [26] to prioritize pathogenic variants based on ACMG guidelines. Secondly, the Franklin (http://franklin.genoox.com) tool was used to select variants based on phenotype according to Human Phenotype Ontology (HPO) terms. Additionally, we performed a target gene investigation considering the panel for primary Immunodeficiency Classification of the International Union of Immunological Societies (IUIS) Expert Committee, updated in 2022 [2]. We identified 116 rare variants classified as pathogenic or likely pathogenic across the 20 patients (see Data Table 3) [27]. Eight heterozygous variants are in genes related to IEI-disorders (IUIS classification) with recessive inheritance pattern according to the Online Mendelian Inheritance in Man (OMIM) database. No compound heterozygous evidence was found. Table 1 provides the links to data file 1, data set 1–2, and data Tables 1, 2 and 3.

**Table 1:** Overview of data files/data tables/data sets

Label	Name of data file/data table/data set	File types (file extension)	Data repository and identifier (DOI or accession number)
Data Set 1	Whole exome sequencing (WES) data from 20 patients with suspicious Inborn errors of Immunity (IEI) manifestation	fastq files (.sra)	NCBI/SRA (https://identifiers.org/ncbi/insdc.sra:SRP411987) [9]
Data Table 1	Demographic characteristics of the cohort	MS Excel file (.xlsx)	Figshare (10.6084/m9.figshare.21674387) [10]
Data Set 2	IEI Exome SNP Discovery	html page (.html)	NCBI/dbSNP (https://www.ncbi.nlm.nih.gov/SNP/snp_viewBatch.cgi?sbid=1063474) [22]
Data Table 2	Overview of the sequencing metrics	MS Excel file (.xlsx)	Figshare (10.6084/m9.figshare.21674435) [23]
Data File 1	Flowchart of the pipeline used to prioritize genetic variants	Image file (.png)	Figshare (10.6084/m9.figshare.21674495) [25]
Data Table 3	Detailed information of the rare and Pathogenic/Likely pathogenic variants found in the cohort	MS Excel file (.xlsx)	Figshare (10.6084/m9.figshare.21674462) [27]

## Limitations

- Absence of clinical and laboratory findings about the 20 patients included in this study.
- Unavailability of molecular and functional studies to validate the variants identified in each patient.
- The limited cohort size to perform population-based studies.
- Lack of investigation of intronic variants or large Structural Variants (SV) limiting our analysis to SNVs and INDELs.

## Data Availability

Data file 1, and Data Tables 1, 2 and 3 described in this Data note can be freely and openly accessible on Figshare (https://figshare.com/) [10, 23, 25, 27]. The raw data of WES dataset (Data Set 1) used in our study is publicly available in SRA-NCBI (https://identifiers.org/ncbi/insdc.sra:SRP411987), SRA accession SRP411987 [9]. The variant data (Data Set 2) generated in this study is publicly available in dbSNP-NCBI (https://www.ncbi.nlm.nih.gov/SNP/snp_viewBatch.cgi?sbid=1063474) [22].

## Abbreviations

ACMG/AMP: American College of Medical Genetics and Genomics and the Association for Molecular Pathology
BQSR: Base Quality Score Recalibration
FIOCRUZ: Fundação Oswaldo Cruz
GATK: Genome Analysis Toolkit
HFL: Hospital Federal da Lagoa
HFSE: Hospital Federal dos servidores do Estado
HPO: Human Phenotype Ontology
IEI: Inborn errors of Immunity
IFF: Instituto Fernandes Figueira
INDEL: Insertion or deletion variants
IPPMG: Instituto de Puericultura e Pediatria Martagão Gesteira
IUIS: International Union of Immunological Societies
NCBI: National Center of Biotechnology Information
OMIM: Online Mendelian Inheritance in Man
SNV: Single nucleotide variant
SRA: Sequence Read Archive
SUS: Sistema Único de Saúde
SV: Structural Variant
UFRJ: Universidade Federal do Rio de Janeiro
WES: Whole exome sequencing
